# Jack Bean (*Canavalia ensiformis*) Tempeh: ACE-Inhibitory Peptide Formation during Absorption in the Small Intestine

**DOI:** 10.17113/ftb.61.01.23.7635

**Published:** 2023-03

**Authors:** Endah Puspitojati, Muhammad Nur Cahyanto, Yustinus Marsono, Retno Indrati

**Affiliations:** 1 Polytechnic of Agricultural Development of Yogyakarta-Magelang, Jl. Kusumanegara, 55167 Umbulharjo, Yogyakarta, Indonesia; 2 Department of Food and Agricultural Product Technology, Faculty of Agricultural Technology, Universitas Gadjah Mada, Jl. Flora, 55281 Bulaksumur, Yogyakarta, Indonesia

**Keywords:** ACE, ACE-inhibitory peptides, small intestine absorption, jack bean tempeh, inhibition pattern

## Abstract

**Research background:**

High blood pressure is the most significant cause of mortality globally. Some fermented foods include ACE-inhibitory peptides that help fight this disease. The ability of fermented jack bean (tempeh) to inhibit ACE during consumption has not been demonstrated yet. This study identified and characterised ACE-inhibitory peptides from jack bean tempeh produced by small intestine absorption using the everted intestinal sac model.

**Experimental approach:**

Sequentially, the protein extract of jack bean tempeh and unfermented jack bean was hydrolysed using pepsin-pancreatin for 240 min. The hydrolysed samples were then evaluated for the peptide absorption using three-segmented everted intestinal sacs (duodenum, jejunum and ileum). The peptides absorbed from all intestinal segments were mixed in the small intestine.

**Results and conclusion:**

The data showed that both jack bean tempeh and unfermented jack bean had the same peptide absorption pattern, with the highest percentage of peptide absorption in the jejunum, followed by the duodenum and ileum. The absorbed peptides of jack bean tempeh exhibited equally strong activity of ACE inhibition in all intestinal segments, while the unfermented jack bean showed strong activity only in the jejunum. The mixture of the peptides from jack bean tempeh absorbed in the small intestine had higher ACE-inhibitory activity (81.09%) than the unfermented jack bean (72.22%). The peptides produced from jack bean tempeh were identified as pro-drug ACE inhibitors and had the mixed inhibition pattern. The mixture of peptides consisted of seven types of peptides with a molecular mass of 826.86–978.20 Da (DLGKAPIN, GKGRFVYG, PFMRWR, DKDHAEI, LAHLYEPS, KIKHPEVK, and LLRDTCK).

**Novelty and scientific contribution:**

This study discovered that consuming jack bean tempeh generated more potent ACE-inhibitory peptides during small intestine absorption than cooked jack beans. Absorbed tempeh peptides have high ACE-inhibitory activity.

## INTRODUCTION

High blood pressure is known as the silent killer as it can cause strokes, coronary infarction, mental degeneration and premature death (*1*). The angiotensin I-converting enzyme (ACE) influences blood pressure regulation in the renin-angiotensin system (RAS). This enzyme converts non-active angiotensin I to active angiotensin II (strong vasopressor) and inhibits the catalytic action of bradykinin (vasodilators), causing artery constriction and elevated blood pressure (*2*). Nowadays, studies about ACE-inhibitory peptides have been extensively reported. These peptides can prevent ACE from converting angiotensin I to angiotensin II (*2*). Based on the changes of the inhibitory action, the ACE-inhibitory peptides are categorised into three types: (*i*) true inhibitors, whose inhibitory activity is stable during digestion, (*ii*) substrate-type peptides, with weak inhibitory activity, and (*iii*) pro-drug peptides, which can be converted to true inhibitors by ACE or digestive tract proteases (*3*). Moreover, only true inhibitors or pro-drug peptides were able to lower systolic blood pressure in hypertensive rats (*3*).

Recently, several studies of food-derived ACE-inhibitory peptides have shifted to many sources of plant proteins (*4*–*6*) which are the sources of protein usually chosen by vegetarians. Jack bean (*Canavalia ensiformis*) is a legume with large seeds that is rarely used as a food source. The seeds have a great potential as a food source since they are rich in proteins and have an adequate amino acid composition (*7*). Previous studies confirm that jack bean is a possible source of ACE-inhibitory peptides (*8*, *9*). The fermentation of jack bean using *Rhizopus oligosporus* for 72 h resulted in the production of peptides with high capacity to inhibit ACE (*9*). Moreover, our previous study revealed that the action of ACE inhibition of both unfermented and fermented jack bean persists after digestion simulation using pepsin pancreatin (*10*).

The fate of food-derived ACE-inhibitory peptides after passing through the gastrointestinal system in the human body is the fundamental issue. The peptides with significant ACE-inhibitory capacity *in vitro* did not always have the same biological activity *in vivo* (*11*). The ACE-inhibitory peptides must be highly stable to maintain their bioactivity until they reach the bloodstream (*12*). Absorption from the digestive tract is critical for allowing ACE-inhibitory peptides to enter the bloodstream and exert their bioactivity (*13*). Study in mice has shown that short peptides, the protein digestion products, were more quickly absorbed in the proximal segment of the small intestine (lower duodenum and upper jejunum). In contrast, some amino acids were more quickly absorbed in the end part of ileum (*14*). Studies about the absorption of ACE-inhibitory peptides have been previously made on koro kratok (*Phaseolus lunatus*) (*6*), koro benguk (*Mucuna pruriens*) (*15*), casein (*16*) and pigeon pea (*Cajanus cajan*) (*17*).

Most previous research has been done to understand the fate of ACE-inhibitory peptide activity after absorption using an everted intestinal model. In contrast, the produced peptides were not identified. This research aims to characterise ACE-inhibitory peptides isolated from jack bean tempeh using the everted intestinal sac model, including their classification, inhibitory pattern, molecular mass and amino acid sequence.

## MATERIALS AND METHODS

### Materials

Jack beans (*Canavalia ensiformis*) were collected from Yogyakarta (Indonesia), tempeh inoculum containing 10^6^ CFU/g of *Rhizopus oligosporus* was obtained from a local market, pepsin (EC 3.4.23.1), pancreatin (EC.232-468-9), angiotensin-converting enzyme (ACE; EC 3.4.15.1), hippuryl-l-histidyl-l-leucine (HHL) were procured from Sigma-Aldrich, Merck (St. Louis, MO, USA), *O*-phthaldialdehyde (OPA) was obtained from Merck (Kenilworth, NJ, USA). The male Sprague-Dawley rats were provided by The Study Center of Food and Nutrition (Universitas Gadjah Mada, Yogyakarta, Indonesia).

### Sample preparation

Jack bean tempeh was prepared according to the method defined by Puspitojati *et al*. (*9*). The seeds were cleaned, steeped for 24 h in water and then cooked for 30 min. After peeling and slicing, the seeds were soaked again in tap water for 48 h. Soaking water was changed every 12 h. After boiling chopped seeds for 30 min, the water was discarded, the seeds were drained and then cooled (30 °C). The cold seeds were inoculated with 0.02% tempeh inocula, mixed, covered with banana leaves and fermented for 72 h (JF72). The unfermented jack bean was used as control (JF0). The resulting product was lyophilised. Both samples were hydrolysed using pepsin (2·10^6^ mU/mL) and pancreatin (10^5^ mU/mL) sequentially at 37 °C for 240 min (*10*). The digestion was simulated according to Minekus *et al.* (*18*) with a slight alteration. The reaction was ended by soaking the solution in 100 °C water for 15 min. The samples were then cooled and centrifuged (5417 R; Eppendorf, Hamburg, Germany) at 8000×*g* and 4 °C for 15 min. The supernatants were taken, lyophilised and stored at −20 °C for further investigation. Each experiment had three replications.

### Preparation of the everted intestinal sacs

Peptide absorption was measured according to the method of Amenta *et al.* (*19*). The Sprague-Dawley male rats (11–12 weeks old, ±250 g) were used. The rats were fasted for 20–24 h and fed water *ad libitum*, then anaesthetised by intramuscular injection using ketamine (180 mg/kg). The experimental animals were placed on the operating table in the supine position. The incision began at the abdomen and proceeded to the left and right sides, cutting the skin and muscle. It then continued towards the cranium and cut the costae to open the thoracic cavity. Then, the intestinal organ was taken and divided into three segments. The duodenum was a U-form section of the intestine located close to the pancreas. The ileum was removed 1 cm from the caecum as the distal part of the small intestine, while the jejunum was segmented between the duodenum and the ileum. The small intestine was everted as soon as possible and placed in 0.9% of sodium chloride. The animal experiment was performed according to the ethics committee approval ref: KE/FK/1384/EC/2018 (Medical and Health Research Ethics Committee (MHREC), Dr. Sardjito General Hospital, Yogyakarta, Indonesia).

### Absorption evaluation

The end of every segment of the small intestine was tied and filled with 1 mL of 0.9% sodium chloride. The peptide samples were put in the tubes. Each small intestinal segment was put into a tube containing a peptide sample. The small intestine must be immersed in a sample-containing mucosal fluid, agitated constantly and oxygenated at 100 bubbles per min. The experiment was performed at 37 °C for 120 min. The sample inside each everted sac was taken and centrifuged (5417 R; Eppendorf) for 15 min at 13 500×*g* and 4 °C. The supernatant obtained from every segment of the small intestine was assayed for ACE-inhibitory action and peptide content. The duodenum, jejunum and ileum supernatants were then mixed for further analysis as the mixed peptides were absorbed in the small intestine.

### Assay of ACE inhibition

The ACE inhibition activity of the absorbed peptides was assayed by a bit of alteration of the Cushman and Cheung method using 8 mmol/L of HHL as the substrate and 25 mU/mL of ACE solution (*20*, *21*). The mixture was mixed for 120 s before being cold centrifuged (5417 R; Eppendorf) for 15 min at 4000×*g*. A volume of 1 mL of the transparent upper layer was collected and dried. A volume of 3 mL of distilled water was used to re-dissolve the residue. The samples were then read at *λ*=228 nm using a UV-Vis spectrometer (Halo SB-10; Dynamica Scientific Ltd., Livingston, UK).

The following equation was used to calculate the ACE-inhibitory activity:


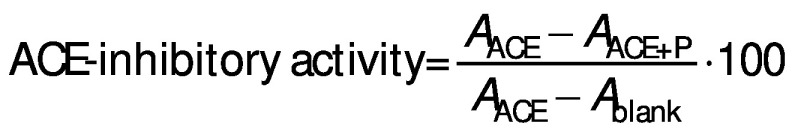
 /1/

where *A*_ACE_ is the absorbance in the presence of ACE, *A*_ACE+P_ is the absorbance in the presence of both ACE and the peptide, and *A*_blank_ is the absorbance of the reaction blank.

### Assay of peptide content

OPA spectrophotometric assay was used for the determination of peptide content (*22*). A mixture of 12.5 mL of 100 mmol/L sodium tetraborate, 1250 µL of 20% sodium dodecyl sulphate and 550 µL of OPA reagent was made. For the OPA-based analysis, 1 mL of OPA and 20 µL of the hydrolysate were mixed together. The mixture was quickly shaken and left to sit for 120 s in the dark, and then the absorbance was measured at *λ*=340 nm using a UV-Vis spectrophotometer (Dynamica Scientific). This assay used tryptone as a standard curve.

### Assay of classification of ACE-inhibitory peptides

A volume of 50 µL of the absorbed peptide sample solution was added to 50 μL of ACE solution (25 mU/mL). The mixture was incubated at 37 °C for 4 h and then 50 μL of HHL solution were added to the mixture. The samples were then incubated for 30 min. The next step followed the ACE-inhibitory activity analysis procedure (*20*).

The peptides were classified according to the difference in their ACE-inhibitory activity before and after ACE incubation. The peptides without significant difference in the inhibitory activity before and after ACE incubation were classified true inhibitors, those with decreased inhibitory activity after ACE incubation as substrate peptides, and the peptides with increased inhibitory activity after the ACE incubation as pro-drug peptides (*3*).

### Evaluation of the pattern of ACE inhibition

The pattern of ACE inhibition was evaluated using the Lineweaver-Burk plot (*23*). The experimental conditions were the same as for the calculation of ACE-inhibitory activity. The activity of the enzyme was calculated using various concentrations of HHL (4, 8 and 12 mM) and inhibitor/peptide concentration (0, 0.25 and 0.5 mg/mL). The type of inhibition was determined by data analysis using the Lineweaver-Burk method to obtain the Michaelis-Menten kinetic constant (*K*_m_). It was calculated based on the regression equation:

y=a+bx /2/

where x is the reciprocal of substrate concentration [1/S] and y is the reciprocal of velocity [1/*v*].

### Determination of amino acid sequences

Amino acid sequences of absorbed peptides were evaluated using ultra-performace liquid chromatograph (UPLC Dionex™ UltiMate 3000 RSLCnano; Thermo Fisher Scientific, Waltham, MA, USA) coupled with high resolution mass spectrometry (HRMS; Q Exactive^TM^, Thermo Fisher Scientific). The absorbed lyophilised peptide was dissolved in 0.1% trifluoroacetic acid (TFA) and adjusted to the final pH<4 of the solution. The peptides were desalted using reversed phase ZipTip C18 pipette tip containing resin (Millipore, Sigma-Aldrich, Merck). ZipTip C18 was placed on a 10-µL micropipette, then moistened using a wetting solution (0.1% TFA in 50% acetonitrile). The wetting solution was aspirated slowly and then discarded slowly. This procedure was repeated three times. Furthermore, ZipTip C18 was equilibrated by aspirating and discarding the equilibration solution (0.1% TFA in water injection) slowly three times. The next step was the binding of the peptide in ZipTip C18. The peptide solution was slowly aspirated and then slowly removed using the same vial. This procedure was conducted repeatedly so that more peptides were bound to the resin. ZipTip C18 was then washed using a washing solution (0.1% TFA in water injection) followed by peptide elution (0.1% TFA in 50% acetonitrile). The eluted peptides were then injected into the instrument. The mobile phase used in this study was 0.01% formic acid in distilled water (nano pump A), 0.1% formic acid in 80% of acetonitrile (nano pump B), and 0.1% formic acid in distilled water (loading pump). The analytical column was EASY-Spray™ column (15 cm×75 µm i.d., PepMap C18; Thermo Fisher Scientific). The obtained data were then identified using Thermo Scientific Proteome Discoverer 2.2 software based on the Sequest HT database (*24*). The results of peptide sequences were then analysed for the potential profile of the biological activity through the BIOPEP analysis (*25*, *26*).

### Statistical analysis

The data were analysed using SPSS v. 23.0 (*27*). One-way ANOVA with a 5% significant difference was used to examine the data. Duncan's multiple range tests assessed the mean differences of the treatments. The *t*-test was performed to compare the data classification of ACE-inhibitory peptides.

## RESULTS AND DISCUSSION

### The absorption of peptides in the small intestine segments

**Fig. 1:**
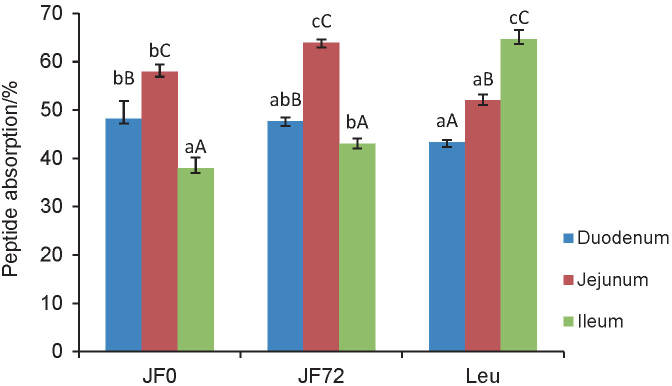
Percentage of absorbed peptides in the small intestine. Mean values with lower-case letters are significantly different in the same segments but in different samples, while those with capital letters are significantly different in the same samples but different segments (p<0.05). JF0=hydrolysate of unfermented jack bean, JF72=hydrolysate of jack bean tempeh fermented for 72 h

The absorption of peptides from hydrolysates of unfermented jack bean (JF0) and tempeh fermented for 72 h (JF72) was investigated in the small intestine using l-leucine as the control. The data showed that the percentage of peptide absorption was significantly different among all intestinal segments in all samples (p<0.05). Fig. 1 shows that JF0 and JF72 have the same peptide absorption pattern, with the highest percentage in jejunum, followed by the duodenum and ileum. In comparison, the highest percentage of absorption of l-leucine was in the ileum, with a value of 64.64%.

The difference in the absorption capacities of peptide samples was speculated due to the different characteristics among duodenum, jejunum and ileum. The jejunum was reported to have a thicker mucosal layer with a larger diameter of 200 million microvilli every 1 mm^2^ to increase the surface area of nutrient absorption by 14- to 40-fold (*28*). These results are also supported by Antunes *et al.* (*29*), who found that the absorption of amino acids as short peptides was faster than of free amino acids. Studies in mice showed that short peptides of protein digestion products were more quickly absorbed in the lower duodenum and upper jejunum. In contrast, some amino acids are more quickly absorbed in the ileum (*14*, *29*).

The percentage of absorbed peptides of JF0 and JF72 in the duodenum was not significantly different (p>0.05), but it was significantly different in the jejunum (p<0.05) and ileum (p<0.05). JF72 exhibited a higher percentage of absorption than JF0 in jejunum and ileum. This was probably due to mould formation during the tempeh fermentation for 72 h, which showed proteolytic activity that could degrade the jack bean protein into shorter peptides and amino acids. Furthermore, fermentation also increased protein solubility (*30*), suggesting that small intestine proteases broke down the substrate more easily to release short peptides and amino acids. Furthermore, JF2 can be absorbed more than JF0. Previous studies reported that the fermentation could improve the digestibility level of legume proteins by eliminating anti-nutrient components that can inhibit the activity of digestive enzymes (*31*). Preliminary studies have shown that JF72 contains more peptides ≤3.5 kDa than JF0. This would probably affect the action of the peptidase enzymes in the brush border membrane. These enzymes can digest the high-molecular-mass peptides slower than smaller peptides that consist of 6-20 amino acids (*28*).

### ACE-inhibitory activity of the absorbed peptides

**Fig. 2:**
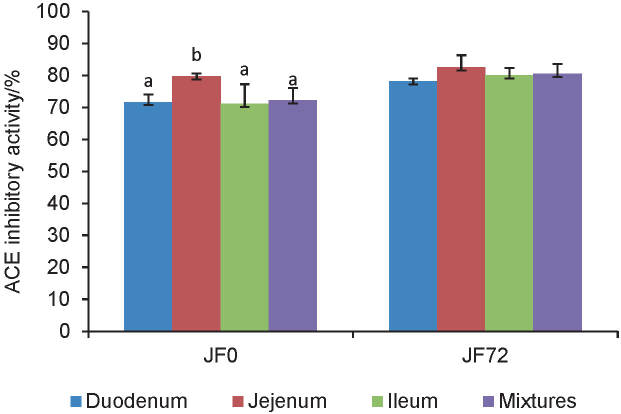
ACE-inhibitory activity of the absorbed peptides. Mean values with different letters are significantly different (p<0.05). Mixture=mixture of peptide solution from duodenum, jejunum and ileum, JF0=hydrolysate of unfermented jack bean, JF72=hydrolysate of jack bean tempeh

Fig. 2 shows the ACE-inhibitory activity of the tempeh peptides that were absorbed by the small intestine. JF0 and JF72 showed significantly different ACE-inhibitory activities in the duodenum, ileum and small intestine. The absorbed peptides in both samples exhibited strong ACE-inhibitory activity. This phenomenon indicated that not all peptides that pass through the brush border membrane were hydrolysed into amino acids and probably there were still short peptides that could be absorbed in the intact form. Some studies stated that di-, tri- and tetrapeptides resistant to hydrolysis of cytoplasmic peptidase could be transported intact to blood circulation (*28*). Fan *et al.* (*32*) added that peptides composed of less than six amino acids could pass through enterocytes with decreasing absorption ability as the chain length increases.

Peptide transport systems were strongly influenced by peptide structure, including chain length, hydrophobicity and charge. Both unfermented and fermented jack beans contained a high concentration of hydrophobic amino acids, including phenylalanine, leucine, proline and isoleucine (*10*). In addition, positively charged amino acids, including arginine and lysine, were found in jack beans. Hydrophobic amino acid residues were reported to be able to strengthen interactions between peptides and dependent peptide transporters (PepT1) (*33*, *34*). Short peptides containing arginine amino acid residues can be transported intact through paracellular diffusion *via* tight junctions (*35*). The resulting peptides produced by the intestinal sac model were speculated to have arginine residue due to the high arginine concentration in the jack bean protein parent.

The best ACE-inhibitory activity of JF0 was obtained in the jejunum at 79.80%. At the same time, JF72 had equally strong ACE-inhibitory activity in all segments of the small intestine (p>0.05). This was thought to be caused by differences in the composition of amino acid of the two raw materials, which caused the difference in the rate of hydrolysis of peptides in the brush border membrane.

Our previous study showed that unfermented jack bean had a higher concentration of leucine, aspartic acid and glutamic acid than jack bean fermented for 72 h (*10*), which probably affected aminopeptidase A, aminopeptidase N and carboxypeptidase G activity in the brush border membrane. Aminopeptidase A was reported to have the specificity to cleave amino acid residues of glutamic acid at the N terminal, whereas aminopeptidase N cleaved alanine residues at the same terminal (*36*). Moreover, carboxypeptidase G was located in the brush border membrane, which had the specificity of cleaving the γ-glutamyl bond at the C terminal. Glutamate carboxypeptidase II in the brush border membrane played a role in the hydrolysis of Asp-Glu and Glu-Glu peptide bonds in the C terminal (*37*). Some endopeptidase enzymes such as meprin αand meprin β also played a role in the hydrolysis of peptides with a specificity of extensive cleaving of hydrophobic amino acid residues (*36*, *37*). The presence of these enzymes was speculated to cause boiled (unfermented) jack bean to undergo hydrolysis more quickly in the jejunum and produce short peptides, which among them have stronger ACE-inhibitory capacity than other segments of the small intestine.

### The classification of ACE-inhibitory peptides of absorbed peptides

**Fig. 3:**
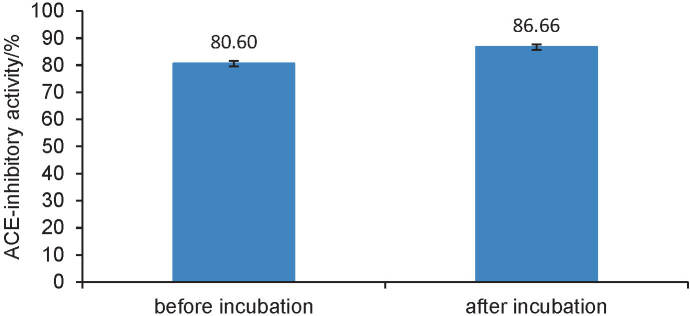
Classification of ACE-inhibitory peptides that are absorbed in the small intestine

Fig. 3 shows that the absorbed peptides (the mixture of peptide solution from duodenum, jejunum and ileum) stimulated a significant increase in the ACE–inhibitory activity after the ACE incubation (p<0.05). As a result of these findings, the peptides might be classed as a pro-drug inhibitors (*3*). There are two speculated causes for the increase in the ACE-inhibitory activity of peptides that have been pre-incubated.

The first possibility is that the short peptides that have residues of amino acids corresponding to the active site of ACE interact with this enzyme. The length of incubation time resulted in more peptides that have the opportunity to interact with the ACE. This condition would result in a higher ACE-inhibitory activity. The second possibility is associated with the broad specificity of ACE, which cleave the dipeptides His-Leu, Tyr-Asp/Glu and Phe-Leu at the C terminal (*1*). The absorbed peptides derived from jack bean tempeh in this study were a mixture of peptides with different chain lengths and amino acid sequences. Some of them might consist of amino acid sequences that fulfil the specificity of ACE cleaving, and hence ACE could hydrolyse the peptides to produce shorter peptides and amino acids. These peptides probably had an excellent affinity to the active site of ACE so the peptides can ultimately inhibit the activity of the ACE.

### ACE inhibition pattern

**Fig. 4:**
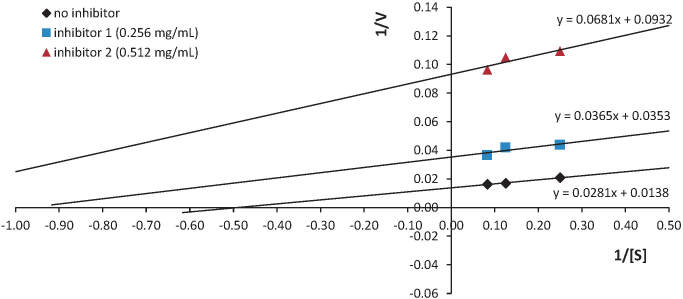
ACE inhibition pattern of the absorbed peptides

The Lineweaver-Burk plot of the pattern of ACE inhibition of the absorbed peptides is given in Fig. 4, where it is observable that the absorbed peptides have a pattern close to uncompetitive inhibition because no intersection points were found on either the x- or y-axis. However, when observing the *v*_max_ and *K*_m_ values in the Lineweaver-Burk plot, it can be seen that the presence of tempeh peptides as ACE inhibitors could reduce *v*_max_ values from 35.59 to 14.68 µmol/min and increase *K*_m_ values from 0.49 to 1.37 (Table 1). The decreasing *v*_max_ and increasing *K*_m_ indicated that the tempeh peptide had a mixed inhibition mechanism (*38*). The pattern of mixed inhibition was indicated by the differences in the small intestine peptide structure. JF72 peptides absorbed in the small intestine still consisted of a mixture of peptides with different characteristics. Peptides that acted as ACE inhibitors also exhibited other inhibitory characteristics due to their affinity for enzymes.

**Table 1:** *v*_max_, *K*_m_ and *K*_i_ of the absorbed peptides

*γ*(inhibitor)/(mg/mL)	A	B	*v*_max_/(µmol/min)	*K*_m_	*K*_i_
0	0.0281	0.0138	35.587	0.491	-
0.256	0.0365	0.0353	27.397	-	0.967
0.512	0.0681	0.0932	14.684	-	1.368

*v*_max_=represents the maximum reaction rate of ACE, *K*_m_=Michaelis-Menten constant, A=1/*v*_max_, B=*K*_m_/*v*_max_

In a mixed inhibition system, the inhibitor does not have the same structure as the substrate but can be bound to free enzymes or the substrate enzyme complex. The mixed inhibition pattern could indicate that the peptides could be attached to the active or inactive site to reduce the catalytic activity of the ACE (*39*). In previous studies, several peptides have been reported to have mixed inhibitory patterns, such as peptide EPNGLLLPQY from walnut protein (*39*) and peptides KAQYPYV, KIIIYN and KILIYG from coconut (*40*).

### Amino acid sequences of the absorbed peptides

The identification of peptides using Thermo Scientific^TM^ Proteome Discoverer v. 2.2 software (*24*) demonstrated that the absorbed peptides consisted of seven types of peptides with molecular mass of 826.86–978.20 Da, with amino acid sequences DLGKAPIN, GKGRFVYG, PFMRWR, DKDHAEI, LAHLYEPS, KIKHPEVK and LLRDTCK. Surprisingly, two of the seven types of produced peptides were fragments of the cowpea severe mosaic virus (CPSMV) polyprotein RNA1. CPSMV is most commonly found in cowpea, but Lima *et al.* (*41*) reported that the virus was also isolated from jack bean. All produced peptides were tested for toxicity prediction using a toxin prediction program (*42*), which revealed that all peptides (JF72) absorbed by the small intestine were non-toxic.

Table 2 shows that the peptides absorbed by the small intestine were short ones consisting of 6-8 amino acids. These results support those of Fernandes-Musoles *et al*. (*35*), who stated that only short peptides resistant to brush border membrane peptidase could be transported intact into the bloodstream. Specific peptides containing 5–9 amino acids, such as RPPGFSPFR, YAEER, KPVAAP and VLPVPQK, have also been shown to be resistant to brush peptidase hydrolysis and capable of being carried intact across monolayers of Caco-2 cell (*33*, *43*, *44*).

**Table 2:** Amino acid sequences of absorbed peptides

Amino acid sequence	*M*/Da	A
DLGKAPIN	826.95	0.500
GKGRFVYG	883.02	0.750
PFMRWR	892.09	0.333
DKDHAEI	826.86	0.149
LAHLYEPS	929.04	0.625
KIKHPEVK	978.20	0.375
LLRDTCK	848.03	0.143

A=frequency of bioactive fragments in a peptide

The peptide mixture absorbed by the small intestine showed ACE-inhibitory activity of 81.09%. According to BIOPEP analysis (*25*, *26*) on the potential profile of its biological activity, all absorbed peptides have the potential as precursors for ACE inhibition. According to the frequency of bioactive fragments in a peptide (A in Table 2), peptides GKGRFVYG, LAHLYEPS and DLGKPIN were speculated to have a substantial role in the strong ACE inhibition in the peptide mixtures. RF, HL and VY were identified as potential ACE inhibitors by molecular docking analysis of jack bean protein (*8*). The three peptide fragments were found in the GKGRFVYG and LAHLYEPS peptides in this study. In the peptide absorption test using the everted intestinal sac method, peptides GKGRFVYG and LAHLYEPS were proven to be resistant to the hydrolysis of peptidase. The small intestine absorbs them in intact form.

## CONCLUSIONS

In an everted intestinal sac model, both unfermented jack bean (JF0) and tempeh (JF72) retained ACE-inhibitory activity after the intestinal absorption. In this study, the peptides of both samples were appropriately absorbed in the small intestine, especially in the jejunum. The jack bean tempeh released seven types of peptides (DLGKAPIN, GKGRFVYG, PFMRWR, DKDHAEI, LAHLYEPS KIKHPEVK and LLRDTCK). The mixture of peptides showed a mixed inhibition pattern with ACE-inhibitory activity of 81.09%. In addition, the released peptides were classified as pro-drug inhibitors.
